# P-223. Changes in body mass index among people with HIV initiating integrase inhibitor based antiretroviral therapy: insights from the TriNetX database

**DOI:** 10.1093/ofid/ofaf695.445

**Published:** 2026-01-11

**Authors:** Athina Schmidt, Laura Mitten, Clara A Chen, Emily Quinn, Gregory Patts, Ivania Rizo, Archana Asundi

**Affiliations:** Boston Medical Center, Boston, Massachusetts; Boston Medical Center, Boston, Massachusetts; Boston University School of Public Health, Boston, Massachusetts; Boston University School of Public Health, Boston, Massachusetts; Boston University School of Public Health, Center for Health Data Science, Boston, Massachusetts; Boston University Chobanian & Avedisian School of Medicine, Boston, Massachusetts; Boston Medical Center, Boston, Massachusetts

## Abstract

**Background:**

Weight gain and obesity among people with HIV (PWH) is an ongoing concern and has been associated in some studies with integrase strand transfer inhibitor (INSTI) antiretroviral (ART) medications. We sought to investigate body mass index (BMI) changes among PWH initiating INSTI vs non-INSTI regimens using the nationwide TriNetX database.

**Table 1:**
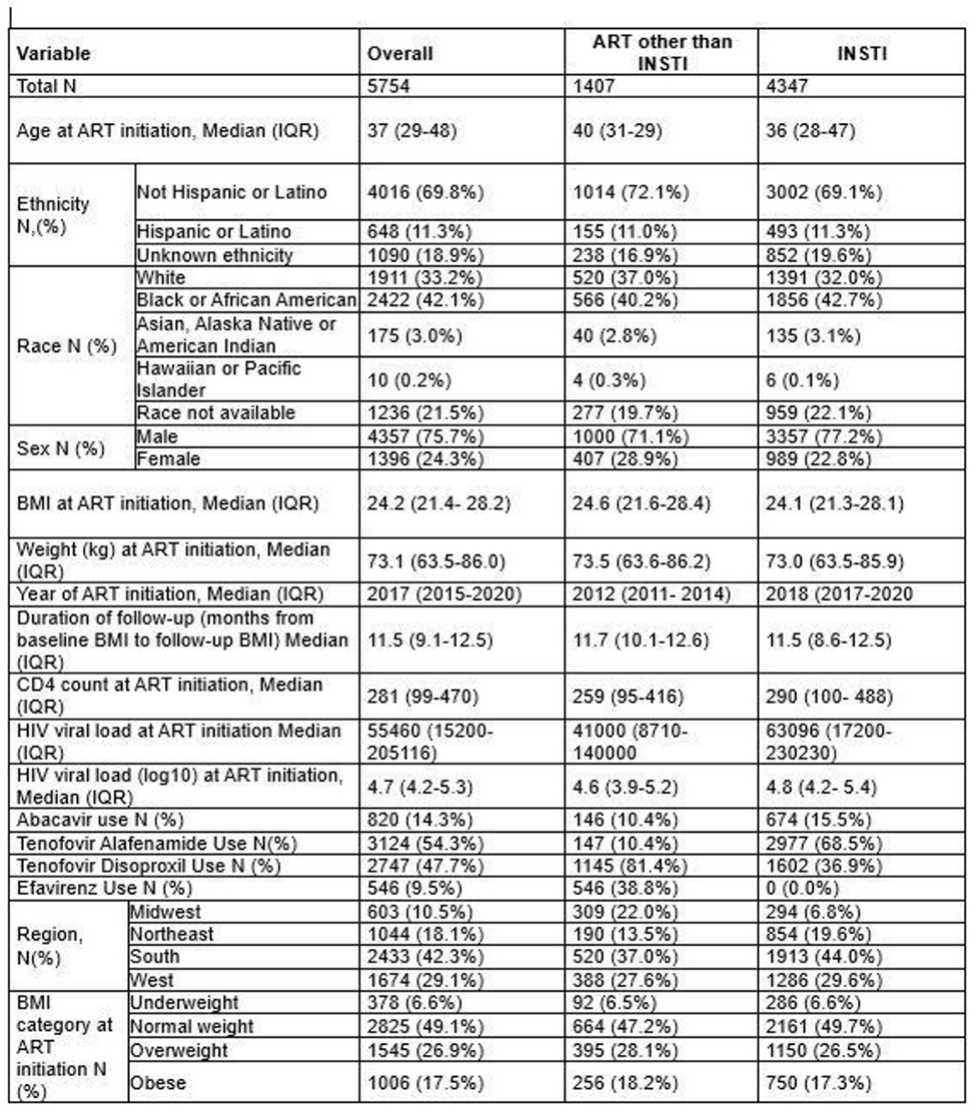
Baseline Characteristics

**Table 2:**
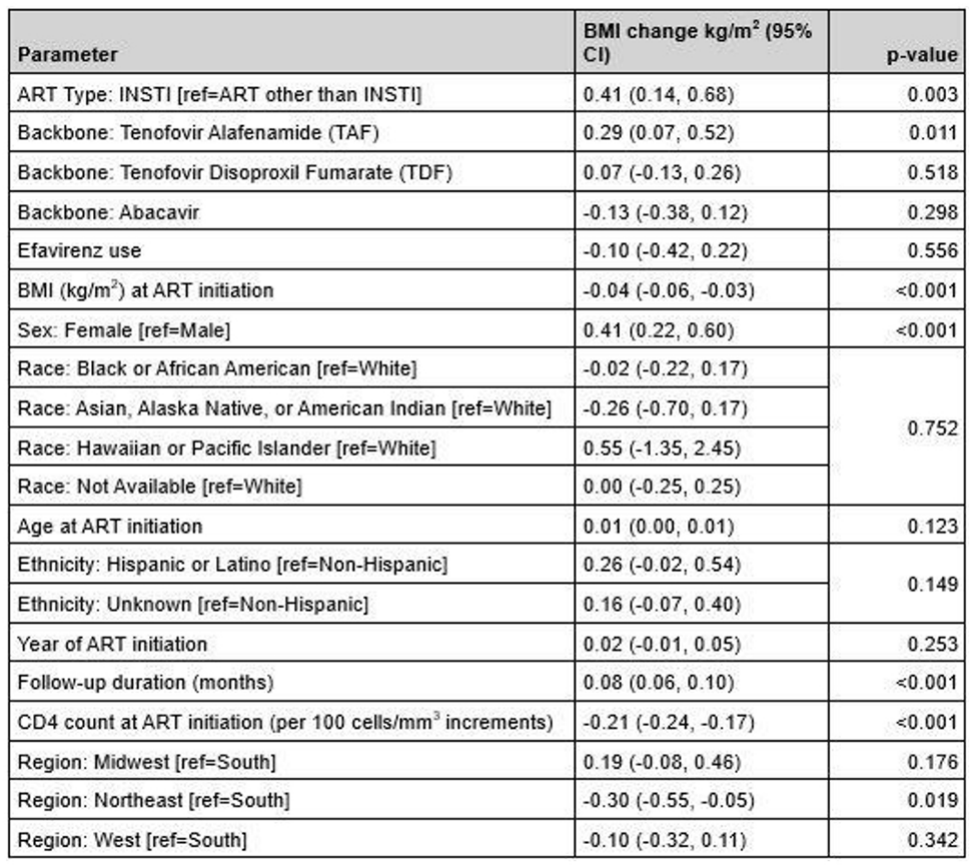
Change in Body Mass Index; Adjusted Model

**Methods:**

TriNetX is a database of electronic medical record data abstracted from over 50 US institutions. Demographic, clinical, and laboratory data were assessed for treatment naive PWH ( >=18y) with available BMI measurements < 30 days from and closest to 12m post ART initiation. We excluded those who switched between INSTI and non-INSTI regimens during follow-up, had mixed-anchor regimens or missing/mishandled data. Bivariate and multivariable generalized linear models were used to assess potential associations between INSTI use and changes in continuous BMI and weight

**Results:**

5754 PWH were included (Table 1); 75.7% male (N=4357) and 42.1% Black (N=2422). Median BMI at ART initiation was 24.2 kg/m^2^ (IQR 21.4-28.2) and median follow-up BMI was 25.5 kg/m^2^ (IQR 22.3-29.8). INSTI (n=4347) and non-INSTI (n=1407) groups differed on median year of ART initiation, median duration of follow-up, median CD4 count at initiation, TAF use and TDF use with some variations in regionality (Table 1). INSTI use was associated with a statistically significant increase in BMI (+0.41kg/m^2^; 95% CI 0.14,0.68, p=0.003) compared to non-INSTIs however no change in BMI category was observed when adjusting for covariates (Table 2). Increased BMI was also associated with baseline BMI, sex, follow-up duration, TAF use, CD4 count at initiation and region. Weight increase for the INSTI group was 6.3% (IQR -0.6-11.7) vs 3.7% (IQR -2.7-8.8) for non-INSTIs.

**Conclusion:**

INSTI use, sex, follow-up duration, and TAF use were associated with small increases in BMI, whereas TDF, EFV, baseline BMI and baseline CD4 were all associated with no change in or decreased BMI, indicating that changes in body weight are multifactorial. Future studies will compare BMI trends to non-HIV populations in order to further factors contributing to weight gain in PWH.

**Disclosures:**

Ivania Rizo, MD, NovoNordisk: Advisor/Consultant Archana Asundi, MD, DayZero Diagnostics: Grant/Research Support|Gilead Sciences: Advisor/Consultant|Gilead Sciences: Grant/Research Support|Theratechnologies: Grant/Research Support|Viiv Healthcare: Grant/Research Support

